# A Pilot Assessment on the Role of Procalcitonin Dynamic Monitoring in the Early Diagnosis of Infection Post Cardiac Surgery

**DOI:** 10.3389/fcvm.2022.834714

**Published:** 2022-06-02

**Authors:** Qiang Miao, Sheng-nan Chen, Hao-jing Zhang, Shan Huang, Jun-long Zhang, Bei Cai, Qian Niu

**Affiliations:** ^1^Department of Laboratory Medicine, West China Hospital of Sichuan University, Chengdu, China; ^2^West China School of Medicine, Sichuan University, Chengdu, China

**Keywords:** procalcitonin, heart surgery, infection, early diagnosis, dynamic monitoring

## Abstract

**Purpose:**

To evaluate the value of dynamic monitoring of procalcitonin (PCT) as a biomarker for the early diagnosis of postoperative infections in patients undergoing cardiac surgery.

**Methods:**

In total, 252 patients who underwent cardiac surgery were retrospectively included. The postoperative patients’ PCT level, change value (△PCT), and clearance rate (△PCTc) were compared between the infected and noninfected groups in adult and pediatric patients on postoperative days (PODs) 1, 3, and 5. The area under the receiver operating characteristic (ROC) curve (AUC) was used to evaluate the diagnostic value.

**Results:**

Procalcitonin concentration decreased progressively in the noninfected group in adult and pediatric patients; PCT concentration continued to rise until it peaked on POD 3 in the infected group. In adult patients, the AUC of PCT for diagnosis of infection on PODs 1, 3, and 5 were 0.626, 0.817, and 0.806, with the optimal cut-off values of 7.35, 3.63, and 1.73 ng/ml, respectively. The diagnostic efficiency of △PCT_3_ and △PCT_*C3*_ was significantly better than △PCT_5_ and △PCT_*C5*_, respectively. In pediatric patients, the AUC of PCT for diagnosis of infection on PODs 1, 3, and 5 were 0.677, 0.747, and 0.756, respectively, and the optimal cut-off values were 27.62, 26.15, and 10.20 ng/ml.

**Conclusion:**

This study showed that dynamic monitoring of PCT levels could be an effective clinical means to help to discover postoperative infection earlier. The PCT level and its change indicators on POD 3 in adult patients and the PCT level on POD 5 in children can indicate infection.

## Introduction

Postoperative infection is one of the most common complications after cardiac surgery. If it is not diagnosed early and treated on time, it will develop into sepsis, prolong the length of hospital stay, increase healthcare costs, and even increase the mortality rate of patients (1, 2). Besides, cardiac surgery under cardiopulmonary bypass (CPB) can induce an inflammatory response of noninfectious origin due to its long duration and significant trauma (3). Both infectious and noninfectious inflammatory responses have the same clinical symptoms, such as fever, leukocytosis, and tachycardia, making the early diagnosis of postoperative infection extremely difficult (4, 5). Therefore, it is crucial to find a biomarker that can distinguish between the two.

Procalcitonin (PCT), which comprises 116 amino acids, is the peptide precursor of calcitonin. It is usually present at undetectable levels in healthy individuals and plays a vital role in predicting infectious inflammatory responses (6, 7). A significantly increased concentration of PCT can be detected when the body suffers from a bacterial infection or sepsis that leads to a severe inflammatory reaction (8). Moreover, generally, its concentration is positively correlated with the severity of the infection (9). Therefore, PCT has been considered a sensitive and specific biomarker for the early diagnosis of sepsis (10, 11). However, in the presence of noninfectious diseases, such as myocardial infarction, malaria, cardiogenic shock from severe traumatic surgery, and burns, the concentration of PCT can also increase, which may be misleading in the diagnosis of early postoperative infection (12). The dynamic monitoring of PCT and the introduction of PCT clearance provide a new direction for early and accurate identification of infections (10, 13, 14). At present, the value of PCT in adults and especially in children after heart surgery is still controversial (15, 16). Therefore, this study aims to evaluate the value of PCT as a biomarker for the early diagnosis of postoperative infections through the dynamic monitoring of PCT levels and clearance after cardiac surgery.

## Materials and Methods

### Subjects

**FIGURE 1:**
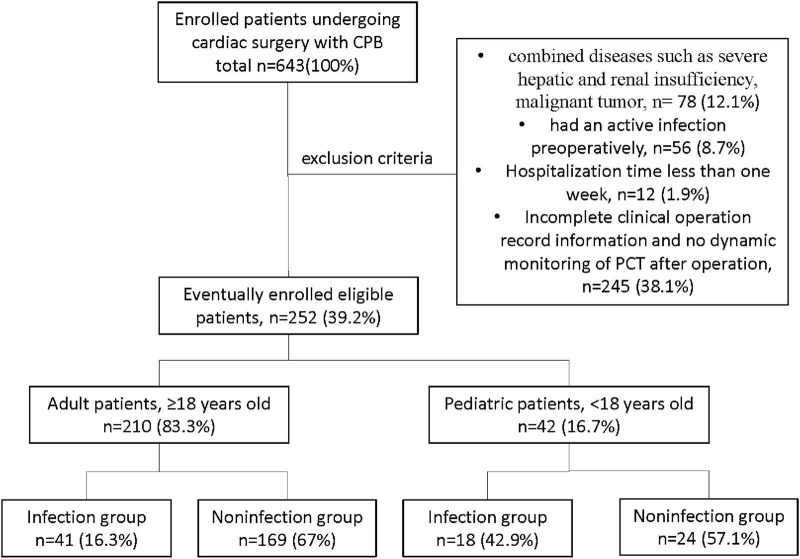
Research object screening.

This retrospective cohort study was performed at the West China Hospital (WCH) of Sichuan University in China. A total of 643 patients from May 2019 to May 2020, with cardiac surgery involving CPB in WCH, were screened for study eligibility, and finally, 252 patients met the following inclusion criteria (Figure 1): (1) those who underwent cardiac surgery involving CPB in WCH, with hospitalization time more than 1 week; (2) those with complete clinical operation record information; (3) dynamic monitoring of PCT was performed after the operation. The other 391 patients met the following exclusion criteria: (1) combined diseases, such as severe hepatic and renal insufficiency, and malignant tumor; (2) had an active infection preoperatively; (3) hospitalization time less than 1 week; (4) with incomplete clinical operation record information and no dynamic monitoring of PCT after surgery. This study protocol was approved by the Ethics Committee of West China Hospital of Sichuan University (No. 2020-823). The data are anonymous, and the requirement for informed consent was therefore waived.

### Study Design

All subjects included in the study were divided into adult (≥ 18 years old) and pediatric (< 18 years old) groups for analysis. The clinical data of patients were collected *via* the Hospital Information System (HIS), including gender, age, CPB time, aortic cross-clamp (ACC) time, hospitalization time, and clinical diagnosis. According to clinical experience and literature reports (17), the vast majority of postoperative infections occur in about 3 days. Hence, serum PCT concentrations on postoperative days (PODs) 1, 3, and 5 were collected *via* the Laboratory Information System (LIS). The presence of postoperative infection was determined according to the patients’ postoperative pathogenic microbiology-related examinations, such as blood, sputum culture and final discharge diagnosis.

### Procalcitonin Related Dynamic Indicators

The concentration of PCT was detected by the Roche Cobas e601 automatic electrochemiluminescence immunoassay analyzer and supporting reagents. This method has been standardized against the BRAHMS PCT LIA assay (Supplementary Material).

△PCT represents the dynamic change value of PCT after cardiac surgery, Which is calculated using the following formula:


(1) PCTPOD⁢ 1-PC⁢TPOD⁢ 3⁢or⁢ 5=△⁢PCT3⁢or⁢ 5

Where POD 1: postoperative day 1; POD 3: postoperative day 3; POD 5: postoperative day 5. △PCT on day 3 (△PCT_3_) and that on day 5 (△PCT_5_) were calculated based on formula (1). PCT clearance (△PCTc) at different time points is calculated using the following formula (10).


(2) (PCTPOD⁢ 1-PCTPOD⁢ 3⁢or⁢ 5)PCTPOD⁢ 1×100%=△PCTPODc⁢ 3⁢or⁢ 5(%)

Where △PCTc on day 3 (△PCTc_3_) and that on day 5 (△PCTc_5_) were calculated based on formula (2).

### Statistical Analysis

Statistical analyses were performed with SPSS 24.0 software (SPSS Inc., Chicago, IL, United States). Measurement data of non-normally distributed variables were described as median (interquartile range, IQR) and compared using the *Mann–Whitney U*-test or *Kruskal–Wallis H*-test. Categorical variables were described as frequencies and percentages, and *Pearson’s chi-square* tests or *Fisher’s* exact tests were performed to analyze the differences between two independent groups. The area under the receiver operating characteristic (ROC) curves (AUC) evaluated the diagnostic efficiency of each variable and performed with the MedCalc^®^ software. The Youden index was used to identify the cut-off values for biomarkers with potential diagnostic significance. Sensitivity, specificity, and optimal cut-off point were calculated following ROC curves for each biomarker. A *p-*value less than 0.05 was considered to be statistically significant.

## Results

### Demographics and Clinical Characteristics

Eventually, a total of 252 patients were included in the study. In which, 210 adult patients consisted of 96 (45.7%) men and 114 (54.3%) women, while the 42 pediatric patients consisted of 22 (52.4%) boys and 20 (47.6%) girls. There were 41 cases in the infectious group and 169 cases in the noninfectious group in the adult patients, and there were 18 cases in the infectious group and 24 cases in the noninfectious group in pediatric patients. The patient’s primary clinical diagnosis includes valvulopathy (24, 11.4%), cardiovascular disease (113, 53.8%), and other heart diseases (73, 34.8%) in adult patients. In comparison, 29 (69%) cases of valvular disease and 31 (31%) cases of other heart diseases were diagnosed in pediatric patients. Table 1 compares the baseline demographic and clinical characteristics of patients. In the adult patients, age and clinical diagnosis were not significantly different between the infectious and noninfectious groups, but a statistically significant gender difference (*p* = 0.001) was found. In the pediatric patients, gender and clinical diagnosis were not significantly different between the infected and noninfected groups, but a significant age difference (*p* = 0.018) was found. Whether in adult or pediatric patients, the length of CPB time, ACC time, and hospital stay in the infected group were significantly longer than those in the noninfected group (all *p* < 0.05, Table 1).

**TABLE 1:** Demographic and clinical characteristics of patients in the noninfected and infected groups.

Variable	Adult patients		*P*	Pediatric patients		*P*
	Infection group (*n* = 41)	Noninfection group (*n* = 169)		Infection group (*n* = 18)	Noninfection group (*n* = 24)	
**Gender**						
Male	28 (68.3%)	68 (40.2%)	0.001	11 (61.1%)	11 (45.8%)	0.327
Female	13 (31.7%)	101 (59.8%)		7 (38.9%)	13 (54.2%)	
Age (Adult: years; Minors: months)	51 (46, 60)	53 (45, 63)	0.491	12 (9, 15)	24 (12, 69)	0.018
**Clinical diagnosis**						
Valvulopathy	3 (7.3%)	21 (12.4%)	0.600	10 (55.6%)	19 (79.2%)	0.113
Cardiovascular disease	22 (53.7%)	91 (53.9%)				
Others	16 (39%)	57 (33.7%)		8 (44.4%)	5 (20.8%)	
Cardiopulmonary bypass time (min)	163 (109.5, 199)	131 (106, 154.5)	0.000	183.5 (124.5, 233)	135.5 (119.3, 174.3)	0.033
Aortic cross clamp (min)	91 (66.5, 110.5)	79 (62, 96)	0.020	108.5 (64.8, 150)	81 (42.8, 109)	0.025
Hospital stay (days)	21 (12, 29)	10 (9, 13)	0.000	46 (22, 53)	13 (8, 18)	0.000

*Data are expressed as n (%) or median (interquartile range).*

### Procalcitonin Concentration and Its Dynamic Changes in the Noninfected and Infected Groups

**FIGURE 2:**
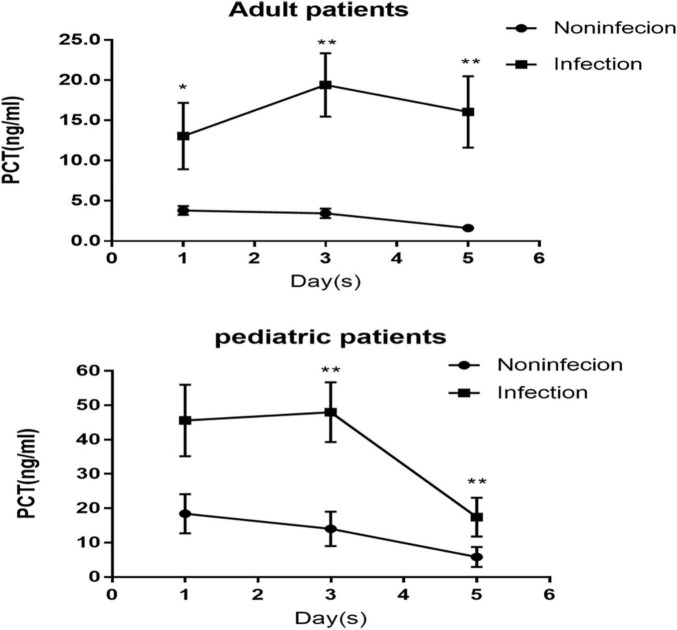
Dynamic changes of procalcitonin (PCT) concentration after cardiac surgery in patients with and without infection.

After CPB, serum PCT concentration decreased progressively in the noninfected group in adult and pediatric patients. In contrast, PCT concentration continued to rise until it peaked on POD 3 in the infected group. In the adult patients, the infected group had a significantly higher PCT level on PODs 1, 3, and 5 (Figure 2). However, lower levels of PCT-related dynamic indicators △PCT_3_, △PCT_5_, △PCT_*C3*,_ and △PCT_*C5*_ (all *p* < 0.05, Table 2) compared with the noninfected group. The PCT level on PODs 3 and 5 in the infected group was significantly higher in the pediatric patients than in the noninfected group (Figure 2). In contrast, the PCT concentration on POD 1 and the levels of PCT-related dynamic indicators △PCT_3_, △PCT_5_, △PCT_*C3*,_ and △PCT_*C5*_ were not significantly different from those in the noninfected group.

**TABLE 2:** Analysis of procalcitonin (PCT) concentration dynamic changes after the operation in the noninfected and infected groups.

Variable	Adult patients		*P*	Pediatric patients		*P*
	Infection Group (*n* = 41)	Noninfection group (*n* = 169)		Infection group (*n* = 18)	Noninfection group (*n* = 24)	
△PCT_3_ (ng/ml)	–2.28 (–1.66, 0)	0.15 (–1.04, 1.24)	0.000	0.16 (–34.88, 12.59)	1.49 (–0.02, 9.11)	0.353
△PCT_5_ (ng/ml)	–0.34 (–3.73, 6.05)	0.32 (–0.38, 2.66)	0.044	11.37 (0.42, 60.08)	5.53 (1.42, 17.4)	0.469
△PCT_*C*3_	–215 (–811.95, 0)	24.06 (–457.890, 50.49)	0.001	11.66 (–158.3, 48.67)	45.24 (–40.27, 63.09)	0.151
△PCT_*C5*_	–28.33 (–309.53, 60.51)	46.25 (–292.56, 78.85)	0.020	62.55 (5.43, 82.6)	72.98 (46.68, 82.94)	0.438

*Data are expressed as median (interquartile range, IQR).*

*PCT_1_: procalcitonin concentration on a postoperative day 1, PCT_3_: procalcitonin concentration on a postoperative day 3, PCT_5_: procalcitonin concentration on a postoperative day 5. △PCT_3_: PCT_1_ minus PCT_3_, △PCT_5_: PCT_1_ minus PCT_5_, △PCT_C3_: △PCT_3_ divided by PCT_1_, △PCT_C5_: △PCT_5_ divided by PCT_1_.*

### Diagnostic Efficacy of Procalcitonin in Postoperative Infection and Poor Prognosis

**FIGURE 3:**
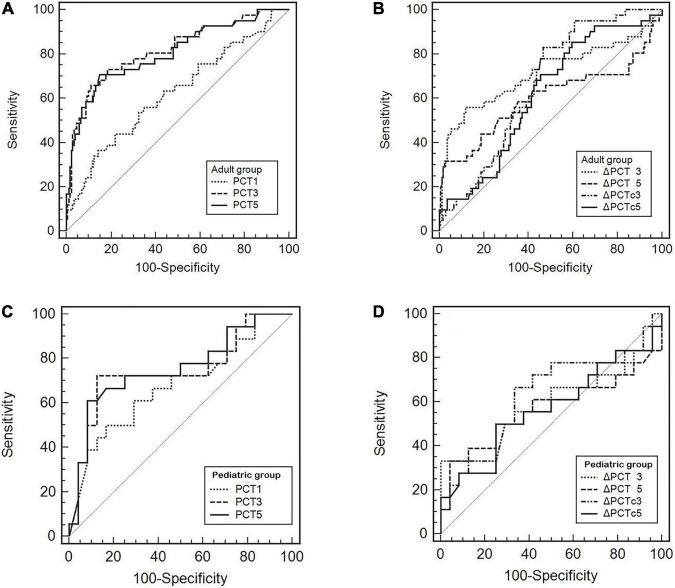
**(A–D)** Receiver operating characteristic (ROC) curves of the PCT level and its related dynamic indicators for predicting postoperative infectious complications.

We further used the ROC curve to analyze the value of PCT levels and related dynamic indicators at different time points after cardiac surgery on postoperative infection. The results are shown in Figure 3 and Table 3. In adult patients, the AUC of PCT for diagnosis of infection on PODs 1, 3, and 5 were 0.626, 0.817, and 0.806, with the optimal cut-off values of 7.35, 3.63, and 1.73 ng/ml, respectively. The diagnostic efficiency of PODs 3 and 5 was significantly higher than POD 1 (*p* < 0.05, Table 3). The AUC of △PCT_3_, △PCT_5_, △PCT_*C3*_, and △PCT_*C5*_ were 0.715, 0.602, 0.663, and 0.617, with the optimal cut-off values were –2.11 ng/ml, –2.11 ng/ml, 16.27%, and 66.32%, respectively. Among them, the diagnostic efficiency of △PCT_3_ and △PCT_*C3*_ were significantly better than △PCT_5_ and △PCT_*C5*_, respectively (*p* < 0.05, Table 3). While in pediatric patients, the AUC of PCT for diagnosis of infection on PODs 1, 3, and 5 were 0.677, 0.747, and 0.756, respectively, and the optimal cut-off values were 27.62, 26.15, and 10.20 ng/ml. The diagnostic efficacy of △PCT_3_, △PCT_5_, △PCT_*C3*,_ and △PCT_*C5*_ were weak, and there is no statistically significant difference compared with the clinical baseline. Unlike adult patients, the PCT level of POD 5 (PCT_5_) in pediatric patients had good diagnostic efficiency for postoperative infection.

**TABLE 3:** Comparison of the diagnostic efficacy of PCT and PCT variation in postoperative infection.

Variable	Adult patients				Pediatric patients			
	AUC (95%CI)	Cut-off value	Sensitivity (%)	Specificity (%)	AUC (95%CI)	Threshold	Sensitivity (%)	Specificity (%)
PCT_1_	0.626^†^ [0.556–0.691]	7.35 ng/ml	36.59	85.80	0.677^†^ [0.515–0.813]	27.62 ng/ml	50.00	83.33
PCT_3_	0.817^†^ [0.758–0.867]*^a^*	3.63 ng/ml	73.17	81.66	0.747^†^ [0.589–0.868]	26.15 ng/ml	72.22	87.50
PCT_5_	0.806^†^ [0.746–0.857]*^a^*	1.73 ng/ml	70.73	85.21	0.756^†^ [0.599–0.875]	10.2 ng/ml	61.11	91.67
△PCT_3_	0.715^†^ [0.649–0.775]*^b^*	–2.11 ng/ml	56.10	88.17	0.584 [0.422–0.734]	–30.92 ng/ml	33.33	100.00
△PCT_5_	0.602 [0.532–0.668]	–2.11 ng/ml	31.71	97.63	0.566 [0.404–0.718]	40.04 ng/ml	33.33	95.83
△PCT_*C*3_	0.663^†^ [0.595–0.727]*^c^*	16.27%	82.93	53.25	0.631 [0.468–0.774]	31.22%	66.67	66.67
△PCT_*C5*_	0.617^†^ [0.547–0.683]	66.32%	85.37	40.24	0.571 [0.409–0.722]	55.14%	50.00	75.00

*^†^Compared to clinical baseline p < 0.05; (a) compared to PCT1 p < 0.05; (b) compared to △PCT_5_ p < 0.05; (c) compared to △PCT_C5_ P < 0.05.*

*PCT_1_: procalcitonin concentration on a postoperative day 1, PCT_3_: procalcitonin concentration on a postoperative day 3, PCT_5_: procalcitonin concentration on a postoperative day 5. △PCT_3_: PCT_1_ minus PCT_3_, △PCT_5_: PCT_1_ minus PCT_5_, △PCT_C3_: △PCT_3_ divided by PCT_1_, △PCT_C5_: △PCT_5_ divided by PCT_1_.*

## Discussion

Cardiac surgery, mainly coronary artery bypass graft and valve disease surgery, is one of the most common surgical procedures. Due to the long operation time, surgical trauma, extracorporeal circulation, hypothermia, aortic clamping, and the presence of multiple drains and catheters, the probability of infections after surgery is significantly increased (3). Early diagnosis and use of appropriate antibiotics to control infection can effectively reduce the mortality rate of postoperative complications, shorten the length of hospital stay, and improve the prognosis of patients undergoing cardiac surgery. PCT is a specific biomarker for the early diagnosis of sepsis and for guiding and evaluating antibiotic therapy efficacy in patients with critical illness (18–20). It was first proposed by s et al. in 1993 that serum PCT could be increased during infection, and the elevation extent of PCT level was related to the severity of infection (21). It has also been reported that PCT was of better specificity and sensitivity in diagnosing bacterial infection when compared with either white blood cell (WBC), C-reactive protein (CRP), or interleukin-6 (IL-6) (22). However, PCT could also be increased due to operation-related factors, such as long-operation time, long-aortic occlusion time, and long-CPB time, even if no infection occurred. Besides, accumulated evidence has shown that compared with the single test value of PCT, the dynamic monitoring of PCT level is of more excellent clinical value for the diagnosis and prognosis of sepsis (15). Facing the difficulty of identifying the presence or absence of an infection early after cardiac surgery, we performed this retrospective pilot assessment to explore whether the dynamic monitoring of PCT had the same value in the early diagnosis of infection after cardiac surgery.

We found that the median age of the infected group was significantly younger than that of the uninfected group among pediatric patients (Table 1), indicating that the younger the children, the more likely they are to be complicated by infection. The possible reason is that newborns are more susceptible to the inflammatory response induced by CPB as the immune system is immature (23). Interestingly, unlike other studies, significant differences in gender between the infected and noninfected groups in adult patients were found in this study. The proportion of men in infected patients is high (Table 1). However, our sample size is relatively small, and the study is a retrospective analysis. So, it is unclear whether this finding has clinical value.

We also found that the PCT levels on PODs 1, 3, and 5 in the infected group were significantly higher than those in the noninfected group among adult patients (Figure 2), and there were also significant differences in the dynamic changes related to indicators △PCT and △PCTc between the two groups (Table 2). These results were consistent with previous studies (3, 6). Compared with adult patients, most pediatric patients are infants, susceptible to complex congenital heart disease and affected by the longer duration of CPB and the more robust response induced by inflammatory CPB. Therefore, compared to the noninfected group among the pediatric patients, only the higher PCT levels on POD 3 and 5 were found in the infected group (Figure 2). In contrast, the levels of PCT on POD 1, △PCT, and △PCTc were not significantly different between the two groups.

It has been shown that PCT, as an indicator of early diagnosis of postoperative infection, may need to be evaluated at different time points and different cut-off values in adult and pediatric patients. We further used the ROC curve to analyze the diagnostic value. We found that the PCT_3_, △PCT_3_, and △PCT_*C3*_ were good values for the diagnosis of infection. The best cut-off value of PCT3 was 3.63 ng/ml for adult patients (Figure 3A and Table 3), which consisted of the previous study findings that the cut-off point to discriminate the presence of infection ranges from 1 to 5 ng/ml (24). Differently, for pediatric patients, we only found that the PCT_5_ has the maximum AUC for the diagnosis of infection. The best cut-off value was also significantly higher than that of adult patients, 10.2 ng/ml (Figure 3C and Table 3). Unfortunately, there was no statistical difference. However, this is consistent with the study by Xia Li et al. (23) who found that the diagnostic properties of PCT could not be observed during the first 3 PODs in pediatric patients. Therefore, more research is needed for pediatric patients to clarify the best time point further and the cut-off value for PCT to predict postoperative infection.

In addition, some studies have reported that PCT plays a specific role in the prognosis evaluation of sepsis and critically ill patients (10, 25). In this study, we found that both adults and children in the infection group had significantly longer hospital stays than those in the noninfection group (Table 1). It suggests that PCT levels may also have a specific value in the prognosis assessment of patients with cardiac surgery.

The limitations should be considered to interpret this study. This was a single-center small sample study, especially for pediatric patients, which may lead to bias in the outcome. Therefore, our results must be further validated by a prospective multi-center study.

## Conclusion

This study showed that dynamic monitoring of PCT levels after cardiac surgery could be an effective clinical means to help discover postoperative infection earlier. The POD 3 PCT level and variation in adult patients could be used to indicate postoperative infection. In contrast, the POD 5 PCT level could potentially indicate a postoperative infection in pediatric patients. Overall, dynamic monitoring of PCT could provide a good reference for clinicians to formulate an effective treatment plan in time.

## Data Availability Statement

The raw data supporting the conclusions of this article will be made available by the authors, without undue reservation.

## Ethics Statement

The studies involving human participants were reviewed and approved by the Ethics Committee of West China Hospital of Sichuan University (No. 2020-823). Written informed consent for participation was not provided by the participants’ legal guardians/next of kin because: The data are anonymous, and the requirement for informed consent was therefore waived.

## Author Contributions

QM: supervision and writing the original draft. S-NC: investigation and data curation. H-JZ: investigation and formal analysis. SH: formal analysis and validation. J-LZ: methodology and supervision. BC: term and conceptualization. QN: project administration and writing and review and editing. All authors reviewed and approved the final manuscript.

## Conflict of Interest

The authors declare that the research was conducted in the absence of any commercial or financial relationships that could be construed as a potential conflict of interest.

## Publisher’s Note

All claims expressed in this article are solely those of the authors and do not necessarily represent those of their affiliated organizations, or those of the publisher, the editors and the reviewers. Any product that may be evaluated in this article, or claim that may be made by its manufacturer, is not guaranteed or endorsed by the publisher.
