# Supplementary material for: A Pilot Assessment on the Role of Procalcitonin Dynamic Monitoring in the Early Diagnosis of Infection Post Cardiac Surgery
Source: Front Cardiovasc Med. 2022 Jun 2;9:834714. doi: 10.3389/fcvm.2022.834714 (PMC9200999; doi:10.3389/fcvm.2022.834714)
Supplement: Supplementary file 1 [file Data_Sheet_1.PDF]

Elecsys BRAHMS PCT

Procalcitonin

REF	Σ	SYSTEM
**05056888** 200	100	Elecsys 2010<br>MODULAR ANALYTICS E170<br>**cobas e** 411<br>**cobas e** 601<br>**cobas e** 602

English

Intended use

Immunoassay for the in vitro quantitative determination of PCT (procalcitonin) in human serum and plasma.

The Elecsys BRAHMS PCT assay can be used to aid in the early detection of clinically relevant bacterial infections.

The **e**lectro**c**hemi**l**uminescence **i**mmuno**a**ssay “ECLIA” is intended for use on Elecsys and **cobas e** immunoassay analyzers.

Summary

Procalcitonin (PCT) is a 116 amino acid prohormone with a molecular weight of approximately 12.7 kD. PCT is expressed by neuroendocrine cells (C cells of the thyroid, pulmonary and pancreatic tissues) and successively enzymatically cleaved into (immature) calcitonin, katacalcin, and an N-terminal region. The blood of healthy individuals contains only low levels of PCT.[1,2] It was discovered that PCT increases during bacterial infection.

It is probable that multiple tissues express PCT throughout the body in response to sepsis as was shown in an animal model.[3] PCT circulating in septic patients consists of only 114 amino acids lacking the N-terminal dipeptide Ala-Pro.[4]

Increased PCT levels are often found in patients suffering from bacterial sepsis, especially severe sepsis and septic shock.[5,6,7,8,9,10] PCT is considered as a prognostic marker to support outcome prediction in sepsis patients.[8,11,12,13]

In acute pancreatitis PCT was found to be a reliable indicator of severity and of major complications.[14,15]

In patients suffering from community-acquired respiratory tract infections or ventilator-induced pneumonia PCT has been proposed as a guide for the decision of antibiotic treatment necessity and to monitor treatment success.[16,17]

Test principle

Sandwich principle. Total duration of assay: 18 minutes.

- 1st incubation: Antigen in the sample (30 µL), a biotinylated monoclonal PCT-specific antibody, and a monoclonal PCT-specific antibody labeled with a ruthenium complex[a)] react to form a sandwich complex.
- 2nd incubation: After addition of streptavidin-coated microparticles, the complex becomes bound to the solid phase via interaction of biotin and streptavidin.
- The reaction mixture is aspirated into the measuring cell where the microparticles are magnetically captured onto the surface of the electrode. Unbound substances are then removed with ProCell/ProCell M. Application of a voltage to the electrode then induces chemiluminescent emission which is measured by a photomultiplier.
- Results are determined via a calibration curve which is instrument-specifically generated by 2-point calibration and a master curve provided via the reagent barcode.

a) Tris(2,2'-bipyridyl)ruthenium(II)-complex (Ru(bpy)$_3^{2+}$)

Reagents - working solutions

The reagent rackpack (M, R1, R2) is labeled as PCT.

M Streptavidin-coated microparticles (transparent cap), 1 bottle, 6.5 mL:

Streptavidin-coated microparticles 0.72 mg/mL; preservative.

R1 Anti-PCT-Ab~biotin (gray cap), 1 bottle, 9 mL:

Biotinylated monoclonal anti-PCT antibody (mouse) 2.0 µg/mL; phosphate buffer 95 mmol/L, pH 7.5; preservative.

R2 Anti-PCT-Ab~Ru(bpy)$_3^{2+}$ (black cap), 1 bottle, 9 mL:

Monoclonal anti-PCT antibody (mouse) labeled with ruthenium complex 5.6 µg/mL; phosphate buffer 95 mmol/L, pH 7.5; preservative.

PCT Cal1 PCT calibrator 1 (white cap), 1 bottle (lyophilized) for 4 mL:

PCT (recombinant) approximately 0.10 ng/mL in a human serum matrix; preservative.

PCT Cal2 PCT calibrator 2 (black cap), 1 bottle (lyophilized) for 4 mL:

PCT (recombinant) approximately 54 ng/mL in a human serum matrix; preservative.

PC PCT1 PreciControl PCT 1 (beige cap), 2 bottles (lyophilized) each for 4 mL:

PCT (recombinant) approximately 0.50 ng/mL in a human serum matrix; preservative.

PC PCT2 PreciControl PCT 2 (brown cap), 2 bottles (lyophilized) each for 4 mL:

PCT (recombinant) approximately 10 ng/mL in a human serum matrix; preservative.

Calibrators: The exact lot-specific calibrator values are encoded in the barcoded labels of the test-specific reagent.

Controls: The exact lot-specific target values and ranges are encoded in the barcodes as well as printed on the enclosed (or electronically available) value sheet.

Precautions and warnings

For in vitro diagnostic use.
Exercise the normal precautions required for handling all laboratory reagents.
Disposal of all waste material should be in accordance with local guidelines.
Safety data sheet available for professional user on request.

This kit contains components classified as follows in accordance with the Regulation (EC) No. 1272/2008:

2-methyl-2H-isothiazol-3-one hydrochloride

EUH 208 May produce an allergic reaction.

Product safety labeling primarily follows EU GHS guidance.

All human material should be considered potentially infectious. All products derived from human blood are prepared exclusively from the blood of donors tested individually and shown to be free from HBsAg and antibodies to HCV and HIV. The testing methods applied were FDA-approved or cleared in compliance with the European Directive 98/79/EC, Annex II, List A.

However, as no testing method can rule out the potential risk of infection with absolute certainty, the material should be handled with the same level of care as a patient specimen. In the event of exposure, the directives of the responsible health authorities should be followed.[18,19]

Avoid foam formation in all reagents and sample types (specimens, calibrators and controls).

Reagent handling

The reagents in the kit (M, R1 and R2) are ready for use and are supplied in bottles compatible with the system.

Calibrators and controls

Carefully dissolve the contents of one bottle by adding exactly 4 mL of distilled or deionized water and allow to stand closed for 15 minutes to

reconstitute. Mix carefully, avoiding foam formation. Transfer the reconstituted calibrators/controls into empty labeled snap-cap bottles.

Unless the entire volume is necessary for calibration and quality control on the analyzer, transfer aliquots of the freshly reconstituted calibrators and controls into empty snap-cap bottles (CalSet Vials/ControlSet Vials). Attach the supplied labels to these additional bottles. Store the aliquots at -20 °C for later use. Perform **only one** calibration or control procedure per aliquot.

Note: Do not combine bottles from different lots. Use only control bottles out of one lot with each other.

All information required for correct operation is read in from the respective reagent barcodes.

Please note: Both the vial labels, and the additional labels (if available) contain 2 different barcodes. The barcode between the yellow markers is for **cobas** 8000 systems only. If using a **cobas** 8000 system, please turn the vial cap 180° into the correct position so the barcode can be read by the system. Place the vial on the instrument as usual.

Storage and stability

Store at 2-8 °C.

Do not freeze.

Store the Elecsys reagent kit **upright** in order to ensure complete availability of the microparticles during automatic mixing prior to use.

Stability of the reagent rackpack	
unopened at 2-8 °C	up to the stated expiration date
after opening at 2-8 °C	12 weeks
on the analyzers	4 weeks

Stability of the calibrators and controls	
lyophilized calibrators/controls	up to the stated expiration date
reconstituted calibrators/controls on the analyzers	2 hours (use only once)
reconstituted calibrators/controls at -20 °C	3 months (freeze only once)

Store the calibrators and controls **upright** in order to prevent the solution from adhering to the snap-cap.

Specimen collection and preparation

Only the specimens listed below were tested in a sufficient number and found acceptable.

Serum collected using standard sampling tubes or tubes containing separating gel.

Li-heparin, K_2-EDTA and K_3-EDTA plasma.

Criterion: Slope 0.9-1.1 + intercept within < ± 2 x analytical sensitivity (LDL) + coefficient of correlation > 0.95.

Stable for 24 hours at 2-8 °C, 3 months at -20 °C. Freeze only once.

After drawing the blood, measure samples within 24 hours or freeze at -20 °C.

Frozen samples can lead to a lower recovery of up to 8 %.

The sample types listed were tested with a selection of sample collection tubes that were commercially available at the time of testing, i.e. not all available tubes of all manufacturers were tested. Sample collection systems from various manufacturers may contain differing materials which could affect the test results in some cases. When processing samples in primary tubes (sample collection systems), follow the instructions of the tube manufacturer.

Centrifuge samples containing precipitates before performing the assay.

Do not use samples and controls stabilized with azide.

Ensure the samples, calibrators and controls are at 20-25 °C prior to measurement.

Due to possible evaporation effects, samples, calibrators and controls on the analyzers should be analyzed/measured within 2 hours.

Materials provided

See “Reagents – working solutions” section for reagents.

- 2 barcode cards
- control barcode sheet
- 2 x 8 bottle labels (calibrators)
- 2 x 14 bottle labels (controls)
- 6 empty labeled snap-cap bottles

Materials required (but not provided)

- REF 11776576322, CalSet Vials, 2 x 56 empty snap-cap bottles
- REF 03142949122, ControlSet Vials, 2 x 56 empty snap-cap bottles
- General laboratory equipment
- Elecsys 2010, MODULAR ANALYTICS E170 or **cobas e** analyzer

Accessories for Elecsys 2010 and **cobas e** 411 analyzers:

- REF 11662988122, ProCell, 6 x 380 mL system buffer
- REF 11662970122, CleanCell, 6 x 380 mL measuring cell cleaning solution
- REF 11930346122, Elecsys SysWash, 1 x 500 mL washwater additive
- REF 11933159001, Adapter for SysClean
- REF 11706802001, Elecsys 2010 AssayCup, 60 x 60 reaction vessels
- REF 11706799001, Elecsys 2010 AssayTip, 30 x 120 pipette tips

Accessories for MODULAR ANALYTICS E170, **cobas e** 601 and **cobas e** 602 analyzers:

- REF 04880340190, ProCell M, 2 x 2 L system buffer
- REF 04880293190, CleanCell M, 2 x 2 L measuring cell cleaning solution
- REF 03023141001, PC/CC-Cups, 12 cups to prewarm ProCell M and CleanCell M before use
- REF 03005712190, ProbeWash M, 12 x 70 mL cleaning solution for run finalization and rinsing during reagent change
- REF 12102137001, AssayTip/AssayCup Combimagazine M, 48 magazines x 84 reaction vessels or pipette tips, waste bags
- REF 03023150001, WasteLiner, waste bags
- REF 03027651001, SysClean Adapter M

Accessories for all analyzers:

- REF 11298500316, ISE Cleaning Solution/Elecsys SysClean, 5 x 100 mL system cleaning solution

Assay

For optimum performance of the assay follow the directions given in this document for the analyzer concerned. Refer to the appropriate operator’s manual for analyzer-specific assay instructions.

Resuspension of the microparticles takes place automatically prior to use. Read in the test-specific parameters via the reagent barcode. If in exceptional cases the barcode cannot be read, enter the 15-digit sequence of numbers.

Bring the cooled reagents to approximately 20 °C and place on the reagent disk (20 °C) of the analyzer. Avoid foam formation. The system automatically regulates the temperature of the reagents and the opening/closing of the bottles.

Place the reconstituted calibrators (in the system-compatible bottles with barcoded labels) in the sample zone.

All the information necessary for calibrating the assay is automatically read into the analyzer.

After calibration has been performed, discard the calibrators.

Analyze the controls PC PCT1 and PC PCT2. The information on the barcoded label of the control serum bottle is read in automatically. After the control procedure has been performed, discard the controls.

Calibration

Traceability: This method has been standardized against the BRAHMS PCT LIA assay.

Every Elecsys BRAHMS PCT reagent set has a barcoded label containing specific information for calibration of the particular reagent lot. The predefined master curve is adapted to the analyzer using PCT Cal1 and PCT Cal2.

Elecsys BRAHMS PCT

Procalcitonin

Calibrator sequence on all systems: Always measure PCT Cal2 before PCT Cal1.

Calibration frequency: Calibration must be performed once per reagent lot using fresh reagent (i.e. not more than 24 hours since the reagent kit was registered on the analyzer). Renewed calibration is recommended as follows:

- after 8 weeks when using the same reagent lot
- after 7 days (when using the same reagent kit on the analyzer)
- as required: e.g. quality control findings outside the defined limits

Quality control

For quality control, use PC PCT 1 and PC PCT 2.

In addition, other suitable control material can be used.

Controls for the various concentration ranges should be run individually at least once every 24 hours when the test is in use, once per reagent kit, and following each calibration.

The control intervals and limits should be adapted to each laboratory's individual requirements. Values obtained should fall within the defined limits. Each laboratory should establish corrective measures to be taken if values fall outside the defined limits.

Follow the applicable government regulations and local guidelines for quality control.

Note: When using two reagent kits with different lots in the same run, the controls will be measured with both reagent lots. Use only control values measured with the corresponding lots.

Calculation

The analyzer automatically calculates the analyte concentration of each sample in ng/mL.

Limitations - interference

The assay is unaffected by icterus (bilirubin < 428 µmol/L or < 25 mg/dL), hemolysis (Hb < 0.559 mmol/L or < 0.900 g/dL), lipemia (Intralipid < 1500 mg/dL) and biotin (< 123 nmol/L or < 30 ng/mL).

Criterion: Recovery within ± 15 % of initial value.

Samples should not be taken from patients receiving therapy with high biotin doses (i.e. > 5 mg/day) until at least 8 hours following the last biotin administration.

No interference was observed from rheumatoid factors up to a concentration of 1500 IU/mL.

There is no high-dose hook effect at PCT concentrations up to 1000 ng/mL.

In vitro tests were performed on 18 commonly used and 10 special pharmaceuticals. No interference with the assay was found.

In rare cases, interference due to extremely high titers of antibodies to analyte-specific antibodies, streptavidin or ruthenium can occur. These effects are minimized by suitable test design.

PCT levels can be increased in certain situations without infectious origin. These include, but are not limited to:[20]

- prolonged or severe cardiogenic shock
- prolonged severe organ perfusion anomalies
- small cell lung cancer or medullary C-cell carcinoma of the thyroid
- early after major trauma, major surgical intervention, severe burns
- treatments which stimulate the release of pro-inflammatory cytokines
- neonates (< 48 h after birth)[21]

For diagnostic purposes, the results should always be assessed in conjunction with the patient's medical history, clinical examination and other findings.

Limits and ranges

Measuring range

0.02-100 ng/mL (defined by the lower detection limit and the maximum of the master curve). Values below the lower detection limit are reported as < 0.02 ng/mL. Values above the measuring range are reported as > 100 ng/mL.

Lower limits of measurement

Lower detection limit of the test

Lower detection limit: ≤ 0.02 ng/mL

The lower detection limit represents the lowest measurable analyte level that can be distinguished from zero. It is calculated as the value lying two standard deviations above that of the lowest standard (master calibrator, standard 1 + 2 SD, repeatability study, n = 21).

Dilution

Samples with PCT concentrations above the measuring range can be diluted manually with PCT negative human serum or plasma. The recommended dilution is 1:4. The concentration of the diluted sample must be > 1.0 ng/mL. After manual dilution, multiply the result by the dilution factor.

Expected values

Reference range

A study performed with the Elecsys BRAHMS PCT assay using 492 samples from apparently healthy males (245) and females (247) revealed the following normal value: 0.046 ng/mL (95th percentile).

Clinical cut-off

Results obtained with the Elecsys BRAHMS PCT assay are in agreement with the literature.[20] A study performed on samples from patients admitted to an ICU (intensive care unit) showed that PCT values:

< 0.5 ng/mL represent a low risk of severe sepsis and/or septic shock

> 2.0 ng/mL represent a high risk of severe sepsis and/or septic shock

Each laboratory should investigate the transferability of the expected values to its own patient population and if necessary determine its own reference ranges.

Clinical performance

Clinical studies were conducted on samples from 283 ICU patients. The patients were classified into categories based on the ACCP/SCCM (American College of Chest Physicians/Society of Critical Care Medicine) consensus criteria on their first day of ICU admission: SIRS (systemic inflammatory response syndrome), sepsis, severe sepsis and septic shock.[22]

The PCT values of the patients with SIRS (n = 95) or sepsis (n = 71) compared to patients with severe sepsis (n = 60) or septic shock (n = 57) were as follows:

Results with a cut-off at 0.5 ng/mL

	Clinical classification		
Elecsys BRAHMS PCT	SIRS	Severe sepsis/ septic shock	Total
< 0.5 ng/mL	63	5	68
≥ 0.5 ng/mL	32	112	144
Total	95	117	212

Based on the above data the sensitivity was 96 %, the specificity 66 %, the positive predictive value 78 % and the negative predictive value 93 %.

	Clinical classification		
Elecsys BRAHMS PCT	SIRS	Sepsis	Total
< 0.5 ng/mL	63	25	88
≥ 0.5 ng/mL	32	46	78
Total	95	71	166

Based on the above data the sensitivity was 65 %, the specificity 66 %, the positive predictive value 59 % and the negative predictive value 72 %.

Results with a cut-off at 2 ng/mL

	Clinical classification		
Elecsys BRAHMS PCT	SIRS	Severe sepsis/ septic shock	Total
< 2 ng/mL	88	18	106
≥ 2 ng/mL	7	99	106
Total	95	117	212

Based on the above data the sensitivity was 85 %, the specificity 93 %, the positive predictive value 93 % and the negative predictive value 82 %.

Elecsys BRAHMS PCT

Procalcitonin

	Clinical classification		
Elecsys BRAHMS PCT	SIRS	Sepsis	Total
< 2 ng/mL	88	55	143
≥ 2 ng/mL	7	16	23
Total	95	71	166

Based on the above data the sensitivity was 23 %, the specificity 93 %, the positive predictive value 70 % and the negative predictive value 62 %.

Specific performance data

Representative performance data on the analyzers are given below. Results obtained in individual laboratories may differ.

Precision

Precision was determined using Elecsys reagents, pooled human serum/plasma and controls in a protocol (EP5-A2) of the CLSI (Clinical and Laboratory Standards Institute): 2 runs per day in duplication each for 21 days (n = 84). The following results were obtained:

Elecsys 2010 and **cobas e** 411 analyzers					
		**Repeatability**		**Intermediate precision**	
Sample	Mean ng/mL	SD ng/mL	CV %	SD ng/mL	CV %
Human plasma 1	0.060	0.005	8.8	0.010	16.3
Human plasma 2	0.622	0.013	2.1	0.026	4.2
Human plasma 3	41.2	0.879	2.1	2.02	4.9
PreciControl PCT1	0.520	0.007	1.3	0.019	3.7
PreciControl PCT2	10.2	0.096	0.9	0.404	4.0

MODULAR ANALYTICS E170, **cobas e** 601 and **cobas e** 602 analyzers					
		**Repeatability**		**Intermediate precision**	
Sample	Mean ng/mL	SD ng/mL	CV %	SD ng/mL	CV %
Human serum 1	0.080	0.006	7.1	0.007	8.7
Human serum 2	0.431	0.008	1.8	0.011	2.6
Human serum 3	54.4	0.618	1.1	0.895	1.6
PreciControl PCT1	0.491	0.013	2.6	0.016	3.2
PreciControl PCT2	9.59	0.181	1.9	0.222	2.3

Method comparison

A comparison of the Elecsys BRAHMS PCT assay (y) with the BRAHMS PCT LIA (x) using human heparin plasma gave the following correlations (ng/mL):

Number of samples measured: 152

Passing/Bablok[23]	Linear regression
y = 1.065x - 0.090	y = 1.143x - 0.194
τ = 0.856	r = 0.981

The sample concentrations were between approximately 0.3 and approximately 82 ng/mL.

A comparison of the Elecsys BRAHMS PCT assay (y) with the BRAHMS PCT sensitive KRYPTOR (x) using human heparin plasma gave the following correlations (ng/mL):

Number of samples measured: 185

Passing/Bablok[23]	Linear regression
y = 0.850x - 0.035	y = 1.090x - 0.709
τ = 0.953	r = 0.988

The sample concentrations were between approximately 0.04 and approximately 85 ng/mL.

Analytical specificity

The Elecsys BRAHMS PCT assay does not show any significant cross reactions with the following substances, tested with PCT concentrations of approx. 0.4 ng/mL and 1.5 ng/mL (max. tested concentration):

Substances	Non-interfering concentrations (ng/mL)
Human katacalcin	30
Human calcitonin	10
Human alpha-CGRP[b)]	10000
Human beta-CGRP	10000

b) Calcitonin Gene-Related Peptide

Functional sensitivity

≤ 0.06 ng/mL

The functional sensitivity is the lowest analyte concentration that can be reproducibly measured with an intermediate precision CV of 20 %.

Concordance with BRAHMS PCT LIA/BRAHMS PCT sensitive KRYPTOR

A comparison study was performed with the Elecsys BRAHMS PCT assay and the BRAHMS PCT LIA. Cut-off values of 0.5 ng/mL and 2 ng/mL have been evaluated.

	BRAHMS PCT LIA		
Elecsys BRAHMS PCT	< 0.5 ng/mL	≥ 0.5 ng/mL	Total
< 0.5 ng/mL	104	49	153
≥ 0.5 ng/mL	6	370	376
Total	110	419	529

	BRAHMS PCT LIA		
Elecsys BRAHMS PCT	< 2 ng/mL	≥ 2 ng/mL	Total
< 2 ng/mL	266	10	276
≥ 2 ng/mL	11	242	253
Total	277	252	529

The concordance between both assays was 90 % at the cut-off value of 0.5 ng/mL and 96 % at the cutoff-value of 2 ng/mL.

The Elecsys BRAHMS PCT assay was also compared to the BRAHMS PCT sensitive KRYPTOR. Cut-off values of 0.5 ng/mL and 2 ng/mL have been evaluated.

	BRAHMS PCT sensitive KRYPTOR		
Elecsys BRAHMS PCT	< 0.5 ng/mL	≥ 0.5 ng/mL	Total
< 0.5 ng/mL	183	20	203
≥ 0.5 ng/mL	2	392	394
Total	185	412	597

	BRAHMS PCT sensitive KRYPTOR		
Elecsys BRAHMS PCT	< 2 ng/mL	≥ 2 ng/mL	Total
< 2 ng/mL	312	24	336
≥ 2 ng/mL	1	260	261
Total	313	284	597

The concordance between both assays was 96 % at the cut-off value of 0.5 ng/mL and 96 % at the cutoff-value of 2 ng/mL.

References

1 Gendrel D, Bohuon C. Procalcitonin as a marker of bacterial infection. Pediatr Infect Dis J 2000;19:679-688.

2 Becker KL, Nylén ES, White JC, et al. Procalcitonin and the Calcitonin Gene Family of Peptides in Inflammation, Infection, and Sepsis: A Journey from Calcitonin Back to Its Precursors. J Clin Endocrinol Metab 2004;89(4):1512-1525.

3 Müller B, White JC, Nylén ES, et al. Ubiquitous Expression of the Calcitonin-I Gene in Multiple Tissues in Response to Sepsis. J Clin Endocrinol Metab 2001;86(1):396-404.

4 Weglöhner W, Struck J, Fischer-Schulz C, et al. Isolation and characterization of serum procalcitonin from patients with sepsis. Peptides 2001;22:2099-2103.

5 Gaïni S, Koldkjær OG, Møller HJ, et al. A comparison of high-mobility group-box 1 protein, lipopolysaccharide-binding protein and procalcitonin in severe community-aquired infections and bacteraemia: a prospective study. Crit Care 2007;11(4):77-87.

6 Castelli GP, Pognani C, Cita M, et al. Procalcitonin, C-reactive protein, white blood cells and SOFA score in ICU: diagnosis and monitoring of sepsis. Minerva Anestesiol 2006;72:69-80.

7 Gaïni S, Koldkjær OG, Pedersen C, et al. Procalcitonin, lipopolysaccharide-binding protein, interleukin-6, and C-reactive protein in community-acquired infections and sepsis: a prospective study. Crit Care 2006;10(2):53-63.

8 Clec'h C, Ferriere F, Karoubi P, et al. Diagnostic and prognostic value of procalcitonin in patients with septic shock. Crit Care Med 2004;32(5):1166-1169.

9 Rey C, Los Arcos M, Concha A, et al. Procalcitonin and C-reactive protein as markers of systemic inflammatory response syndrome in critically ill children. Intensive Care Med 2007;33:477-484.

10 Andreola B, Bressan S, Callegaro S, et al. Procalcitonin and C-Reactive Protein as Diagnsotic Markers of Severe Bacterial Infections in Febrile Infants and Children in the Emergency Department. Pediatr Infect Dis J 2007;26(8):672-677.

11 Novotny A, Emmanuel K, Matevossian E, et al. Use of procalcitonin for early prediction of lethal outcome of postoperative sepsis. The American Journal of Surgery 2007;194:35-39.

12 Hausfater P, Juillien G, Madonna-Py B, et al. Serum procalcitonin measurement as diagnostic and prognostic marker in febrile adult patients presenting to the emergency department. Crit Care 2007;11(3):60-69.

13 Dahaba AA, Hagara B, Fall A, et al. Procalcitonin for early prediction of survival outcome in postoperative critically ill patients with severe sepsis. Br J Anaesth 2006;97:503-508.

14 Rau B, Schilling MK, Beger HG. Laboratory Markers of Severe Acute Pancreatitis. Dig Dis 2004;22:247-257.

15 Sato N, Endo S, Kasai T, et al. Relationship of the serum procalcitonin level with the severity of acute pancreatitis. Research Communications in Molecular Pathology and Pharmacology 2004;115,116:243-249.

16 Stolz D, Christ-Crain M, Gencay MM, et al. Diagnostic value of signs, symptoms and laboratory values in lower respiratory tract infection. Swiss Med Wkly 2006;136:434-440.

17 Christ-Crain M, Müller B. Biomarkers in respiratory tract infections: diagnostic guides to antibiotic prescription, prognostic markers and mediators. Eur Respir J 2007;30:556-573.

18 Occupational Safety and Health Standards: bloodborne pathogens. (29 CFR Part 1910.1030). Fed. Register.

19 Directive 2000/54/EC of the European Parliament and Council of 18 September 2000 on the protection of workers from risks related to exposure to biological agents at work.

20 Meisner M. Procalcitonin (PCT) – A new innovative infection parameter. Biochemical and clinical aspects. Thieme Stuttgart, New York 2000, ISBN: 3-13-105503-0.

21 Chiesa C, Panero A, Rossi N, et al. Reliability of Procalcitonin Concentrations for the Diagnosis of Sepsis in Critically ill neonates. Clin Infect Dis 1998;26:664-672.

22 American College of Chest Physicians/Society of Critical Care Medicine Definitions for sepsis and organ failure and guidelines for the use of innovative therapies in sepsis. Crit Care Med 1992;20:864-874.

23 Bablok W, Passing H, Bender R, et al. A general regression procedure for method transformation. Application of linear regression procedures for method comparison studies in clinical chemistry, Part III. J Clin Chem Clin Biochem 1988 Nov;26(11):783-790.

This product may not be used by purchaser to conduct any Point-of-Care testing including but not limited to any near patient testing on hospital wards and/or emergency departments and/or in doctor's offices and/or in any other locations outside private or public clinical laboratories. No general patent or other license of any kind other than this specific right of use from purchase is granted hereby.

For further information, please refer to the appropriate operator's manual for the analyzer concerned, the respective application sheets, the product information and the Method Sheets of all necessary components (if available in your country).

A point (period/stop) is always used in this Method Sheet as the decimal separator to mark the border between the integral and the fractional parts of a decimal numeral. Separators for thousands are not used.

Reagent developed in collaboration with B·R·A·H·M·S.

B·R·A·H·M·S PCT is a registered trademark of BRAHMS Aktiengesellschaft.

B·R·A·H·M·S PCT ELECSYS

Symbols

Roche Diagnostics uses the following symbols and signs in addition to those listed in the ISO 15223-1 standard.

CONTENT	Contents of kit
SYSTEM	Analyzers/Instruments on which reagents can be used
REAGENT	Reagent
CALIBRATOR	Calibrator
→	Volume after reconstitution or mixing
GTIN	Global Trade Item Number

COBAS, COBAS E, ELECSYS, MODULAR and PRECICONTROL are trademarks of Roche. INTRALIPID is a trademark of Fresenius Kabi AB.

All other product names and trademarks are the property of their respective owners.

Significant additions or changes are indicated by a change bar in the margin.

© 2014, Roche Diagnostics

Roche Diagnostics GmbH, Sandhofer Strasse 116, D-68305 Mannheim
www.roche.com

Roche
